# Immune Impairment Associated with Vitamin A Deficiency: Insights from Clinical Studies and Animal Model Research

**DOI:** 10.3390/nu14235038

**Published:** 2022-11-26

**Authors:** Joshua O. Amimo, Husheem Michael, Juliet Chepngeno, Sergei A. Raev, Linda J. Saif, Anastasia N. Vlasova

**Affiliations:** 1 Center for Food Animal Health, Department of Animal Sciences, College of Food Agricultural and Environmental Sciences, Ohio State University, 1680 Madison Avenue, Wooster, OH 44691, USA; 2 Department of Animal Production, Faculty of Veterinary Medicine, University of Nairobi, P.O. Box 29053, Nairobi 00625, Kenya

**Keywords:** vitamin A deficiency, immune responses, gut microbiome, infectious diseases, vaccines

## Abstract

Vitamin A (VA) is critical for many biological processes, including embryonic development, hormone production and function, the maintenance and modulation of immunity, and the homeostasis of epithelium and mucosa. Specifically, VA affects cell integrity, cytokine production, innate immune cell activation, antigen presentation, and lymphocyte trafficking to mucosal surfaces. VA also has been reported to influence the gut microbiota composition and diversity. Consequently, VA deficiency (VAD) results in the imbalanced production of inflammatory and immunomodulatory cytokines, intestinal inflammation, weakened mucosal barrier functions, reduced reactive oxygen species (ROS) and disruption of the gut microbiome. Although VAD is primarily known to cause xerophthalmia, its role in the impairment of anti-infectious defense mechanisms is less defined. Infectious diseases lead to temporary anorexia and lower dietary intake; furthermore, they adversely affect VA status by interfering with VA absorption, utilization and excretion. Thus, there is a tri-directional relationship between VAD, immune response and infections, as VAD affects immune response and predisposes the host to infection, and infection decreases the intestinal absorption of the VA, thereby contributing to secondary VAD development. This has been demonstrated using nutritional and clinical studies, radiotracer studies and knockout animal models. An in-depth understanding of the relationship between VAD, immune response, gut microbiota and infections is critical for optimizing vaccine efficacy and the development of effective immunization programs for countries with high prevalence of VAD. Therefore, in this review, we have comprehensively summarized the existing knowledge regarding VAD impacts on immune responses to infections and post vaccination. We have detailed pathological conditions associated with clinical and subclinical VAD, gut microbiome adaptation to VAD and VAD effects on the immune responses to infection and vaccines.

## 1. Introduction

**Figure 1:**
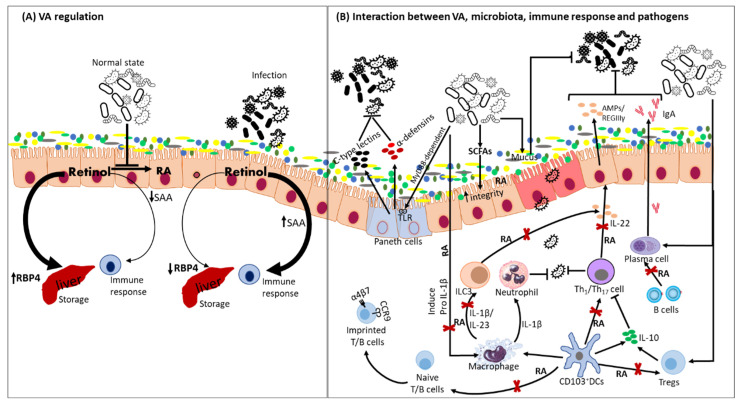
Multidirectional relationship between VA, gut microbiome, immune response and infections. (**A**) In normal state, the beneficial gut microbiome promotes RA storage via suppressing conversion of dietary retinol into retinoic acid (RA), hence increasing retinol transporter retinol binding protein 4 (RBP4) and reducing acute-phase RBPs (serum amyloid A (SAA)). However, during infection, RBP4 amounts decrease, resulting in low circulating retinol and low hepatic storage, while SAA levels increase, leading to upregulation of local immune response to infection. (**B**) Beneficial microbes upregulate the host epithelial barrier function by production of mucus and short-chain fatty acids (SCFAs), and the microbiota stimulates secretion of IL-1β, leading to elevated neutrophil recruitment to infection site, promoting secretion of IL-22 and resulting in release of antimicrobial peptides. Commensals also act through MyD88-dependent mechanism to trigger production of antimicrobials (AMPs) such as α-defensins, C-type lectins and REGIII-γ by Paneth cells. Beneficial microbes promote adaptive immunity by stimulating Th1 and Th17 cells, leading to increased production of IL-22. Finally, the microbiome prevents pathogen colonization by regulating the secretion of IgA by plasma cells and differentiation of T regulatory cells. VA through its metabolite RA is essential in strengthening host–barrier integrity, enhancing innate immune response and modulating adaptive immunity (effect indicated by arrows). Following antigen stimulation, CD103^+^DCs in gut-associated lymphoid tissues (GALT) produce RA that imprints gut-homing specificity on T and B cells expressing both α4β7 and CCR9, which migrate to small intestine tissues and bind to MAdCAM-1 and CCL25, respectively. The X (red) in the arrows indicates immune response pathways affected when the host is deficient in VA (VAD).

Vitamin A is a fat-soluble vitamin obtained primarily from the diet. Dietary sources of VA include organ meats such as liver, milk and provitamin A carotenoids from vegetables and fruits [1]. VA is found in the form of retinol, retinal and retinoic acid (RA), of which RA is the most important biologically active metabolite. Major VA functions include the maintenance of vision, growth and development and the integrity of epithelial and mucosal tissues [2,3]. However, multiple studies have shown that major immune organs require a steady dietary intake of VA to maintain their functions well [4]. Previous research has described the complexity of the mechanisms through which VA influences immune responses in the host (Figure 1). For example, VA status affects cell integrity, cytokine secretion, the regulation of innate and adaptive immunity, antigen presentation and lymphocyte homing to mucosal surfaces (Figure 1) [5]. Additionally, VA regulates the antimicrobial activity of macrophages, which leads to phagocytic and oxidative burst activity [6,7]. In addition to the direct influence on the immune response, VA status has been associated with the regulation of gut microbiome composition [8,9]. On the other hand, the gut microbiota has been shown to be involved in priming and modulating mucosal and systemic immunity as shown in Figure 1. Thereby, the perturbation of the gut microbiome leads to dysbiosis, the underrepresentation of beneficial species and the overgrowth of pathobionts.

Given the above important roles of VA as shown in Figure 1, its deficiency (VAD) is known to impair all the pathways that are dependent on RA for the effective induction of host immune responses. VAD has indeed been associated with the exacerbation of many infectious diseases, dysbiosis and a reduced immune response to vaccines. VAD is linked to numerous pathological conditions including immune disorders, bone abnormalities, impaired vision and compromised epithelial barriers of mucosal surfaces. These pathological processes are major contributors to the increased risk of infection in VAD individuals. Mechanisms through which VAD occurs in individuals have been investigated in humans and animals using nutritional, clinical and experimental (VA deprivation) studies (in humans, pigs, calves, rodents, chicken, monkeys), knockout (retinol binding protein (RBP), retinoic acid (RA) receptors (RAR, RXR), lecitihin: retinol acyltransferase (LRAT)) animal models (rats, mice) and radiotracer (3H-retinol or 14C-RA) studies (mice). From these studies, VAD has been associated with impaired functions of macrophages, neutrophils and natural killer (NK) cells and diminished protective function at the mucosal surfaces [10]. VAD resulted in increased macrophage-mediated inflammation through the enhanced production of interleukin 12 (IL-12) and interferon gamma (IFN-γ) cytokines and the reduced phagocytic capacity of macrophages that thereby increased pathogen replication at the site of infection and exacerbated inflammation [11,12]. Moreover, VAD has been associated with decreased secretory immunoglobulin A (sIgA) levels due to the reduced transport of mucosal immunoglobulin A (IgA) to epithelial surfaces, while VA supplementation increased the concentrations of IgA in VAD mice [13,14,15]. Additionally, VAD is correlated with dysregulated adaptive immune responses where it increases CD8^+^ T cell numbers, IFN-α levels and proinflammatory IL-12 levels while reducing anti-inflammatory IL-10 levels in gnotobiotic (Gn) pigs [16].

VAD remains a major public health challenge, predominantly in developing countries, as well as in low-income settings of developed nations, and it is a substantial contributor to morbidity and mortality caused by infections, especially in children, elderly individuals and pregnant women. The World Health Organization (WHO) has listed VAD as one of the leading micronutrient deficiencies affecting children and women mostly in developing countries [17]. Generally, pregnant and breastfeeding females are vulnerable to VAD. Normal retinol concentrations range between 0.52 and 2.09 mmol/L; however, plasma or serum retinol concentration < 0.70 μmol/L signifies subclinical VAD in children and adults, and a concentration of <0.35 µmol/L indicates severe VAD. A study in Brazil reported an association of VAD with lower intake of retinol, underweight children and adolescents. Although VAD seems not to have a gender predilection, retinol activity equivalents (RAE) in males (900 µg) tend to be higher than in females (700 µg). In this review, we present a comprehensive discussion and analysis of the current literature regarding the causes and pathology associated with VAD and find that VAD affects immune response to infectious diseases and vaccines. We also summarize the evidence of VAD influence on the gut microbiota composition and diversity, providing insights from clinical studies and animal model research. In-depth knowledge of the impact of VAD on interactions between VA, gut microbiome and immune responses during infections and in healthy individuals will aid in optimizing vaccine performance and developing effective strategies to prevent and treat infectious diseases in countries where VAD is prevalent.

## 2. Vitamin A Deficiency and the Associated Pathology

### 2.1. How Vitamin A Deficiency Occurs

**Figure 2:**
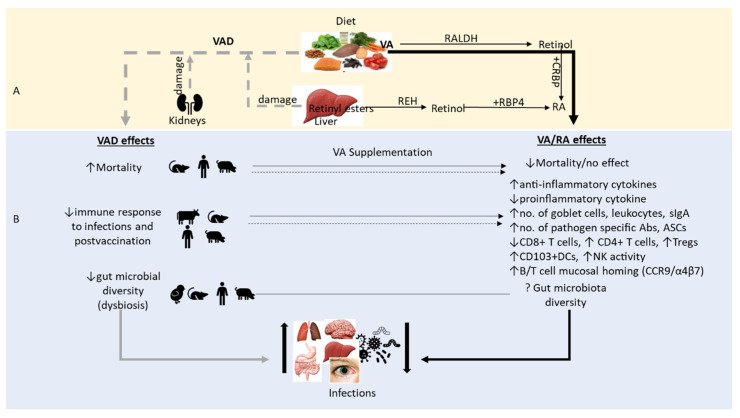
(**A**) Vitamin A (VA) metabolism and factors contributing to VA deficiency (VAD). (**B**) VA supplementation to VAD hosts reversed the negative impact of VAD on immune system and gut microbiota. Thin solid line with arrow—vitamin A supplementation (VAS) rescued effects of VAD; thin dotted line—VAS did not rescue the effects of VAD in clinical studies; thin solid line without arrow/?—VAS effects not known. RALDH, retinaldehyde dehydrogenase; REH, retinyl ester hydrolase; CRBP, cellular retinol-binding protein; ASCs, antibody secreting cells.

Vitamin A is a key dietary micronutrient for the well-being, health and survival of humans and animals since it is critical for numerous physiological processes, including the maintenance of vision, growth, reproduction and the integrity of epithelial and mucosal tissues and the regulation of the immune system [2,3]. Mechanisms through which VAD occurs have been investigated in humans and animals using nutritional, clinical and experimental (VA deprivation) studies, knockout animal models and radiotracer studies. The primary cause of VAD is consuming a diet low in VA; however, diseases or conditions that interfere with VA absorption from the intestines, such as chronic diarrhea, celiac disease and pancreatic disorders as well as pathologies causing kidney and liver disorders also contribute to VAD (Figure 2A). For example, VAD can result from the excessive loss of VA in urine due to pathologies associated with kidney dysfunction or acute kidney injury such as chronic kidney disease, sepsis and amyloidosis [18]. Studies have demonstrated that the interactions between different components of retinol transport (RBP4 and transthyretin) play key roles in VA homeostasis, and the excessive loss of retinol-bound RBP4 in the kidneys, usually associated with transthyretin, may lead to VAD [19,20,21]. Secondly, conditions causing a reduction in RA receptor alpha (RXRα) expression may also lead to VAD. RXRα plays a critical role in the regulation of macrophage immune function; for instance, abnormalities in their function can result in dysregulated immune responses. Chow and coauthors demonstrated that RXRα expression is inhibited by vesicular stomatitis virus-activated interferon regulatory factor 3 (IRF3) [22]. Thus, even in the presence of physiologically normal serum/liver VA levels, VA function may be affected due to the downregulation of RXRα expression during viral infections. Thirdly, when there is reduced dietary VA intake, retinyl esters stored in the liver are converted to retinol by retinyl ester hydrolase and released back into circulation. VAD results in the downregulation of lecitihin: retinol acyltransferase (LRAT) expression in the liver, thereby increasing the hydrolysis of retinyl ester, releasing free retinol from the hepatic reserves. Moreover, VAD stimulates the expression of RBP; hence, more RBP is available and ready to bind to retinol. As long as there is insufficient dietary VA intake, the liver continues to mobilize its VA stores to support other extrahepatic tissues. However, as hepatic VA levels are depleted, the serum retinol concentrations eventually drop, and the clinical signs of VAD appear [23]. As the severity of VAD increases, its clinical and biochemical manifestations, of which some are reversible, contribute to the vicious cycle of VAD. Further, RA itself has been found to regulate hepatic LRAT activity, as was shown in a study in which supplementation with RA (not retinol) restored LRAT activity within 12–16 h [24]. This mechanism is essential in maintaining a constant level of circulating retinol, as was demonstrated in VAD rats fed a VA-deficient diet, where they retained normal levels of VA in the circulation for 84 days [23]. Infectious and noninfectious diseases that cause liver damage such as cystic fibrosis, bile duct blockage and liver cirrhosis may interfere with this mechanism, thereby exacerbating VAD. Grumet and coworkers in their review summarized pathological conditions that lead to retinyl ester mobilization rather than its storage in liver [25]. However, in contrast, other liver diseases, such as nonalcoholic fatty liver disease, were associated with enhanced retinol storage in liver, although mechanisms behind this phenomenon are unclear [26,27].

### 2.2. Pathologies Associated with VAD 

Pathological conditions associated with VAD are numerous including immune disorders, bone abnormalities, impaired vision and compromised epithelial barriers of mucosal surfaces. These pathological processes are major contributors to the increased risk of infection in VAD individuals, although infection itself can exacerbate VAD. The most recognized clinical manifestation of VAD is xerophthalmia, a progressive eye disease initially presenting as dry and crusted eyes that can develop into night blindness, conjunctival xerosis, corneal ulcers and Bitot’s spots [28,29]. VA has also been shown to be essential in the creation and repair of skin cells in cases of skin infections; thus, VAD may present as skin irritation. VA aids in the formation of collagen (an essential part of healthy skin), critical for wound healing. Studies of rats and humans have demonstrated poor wound healing in VAD hosts and confirmed that VA treatment enhanced wound healing or prevented wound development [30,31]. Additionally, studies have shown that VA supplementation (VAS) reduces skin inflammation caused by eczema [32,33,34].

Since VA regulates the differentiation of epithelial tissues and prevents the keratinization of mucosal surfaces, VAD causes pathological changes in the epithelial layer, resulting in stratified keratinized epithelium and damaged ciliated cells, in turn resulting in reduced mucus production and goblet cells [35]. Recent studies have shown that VAD causes keratinizing metaplasia of the epithelial barrier, leading to urinary and respiratory tract infections. The combined effects of VAD and respiratory infections resulted in the development of squamous metaplasia in the lung, increasing risk of suffering respiratory conditions including asthma [36,37,38,39]. Similar changes in squamous cells were observed in VAD mice infected with influenza virus [40]. These abnormalities of the mucosal airways weaken pulmonary defenses against pathogens, hindering their clearance from the respiratory tract and upregulating mucosal cytokines [41]. Furthermore, Fan and coauthors demonstrated impaired mucin expression that suppressed the mucosal immune function (decreased IgA) of the airways in VAD chicks [42]. VAD causes massive damage to the mucosa of the gastrointestinal tract, affecting mucous layer homeostasis and resulting in reduced mucin (MUC) 2 and elevated MUC3 expression, lower α-defensin 6 production and upregulated toll-like receptor (TLR) 2 and 5 expression [43]. Blunting of villi tips observed in VAD mice was associated with decreased numbers of goblet cells [44]. Liang and coauthors demonstrated that VAD causes intensified neurological tissue damage in VAD mice infected with lymphocytic choriomeningitis virus leading to hyperreactive T cell responses [45].

VA is essential in regulating the population of myeloid cells and providing homeostasis in bone marrow, spleen and peripheral blood [46]. Studies have reported a decrease in weight resulting in small germinal centers and the cellularity of the spleen of VAD rats [47]. Similarly, studies have shown that VAD leads to atrophic changes in lymphoid tissues including thymus weight and atrophy in rats [44] and the involution of thymus and bursa of Fabricius in chickens [45], resulting in decreased numbers of lymphocytes in the circulation. However, in mice, VAD alone had no effect on spleen size, but VAD accompanied with inanition resulted in spleen enlargement. VAD has also been associated with the enlargement of local lymph nodes in rats and mice, probably due to the accumulation of cell debris and the cellular composition [11,48,49]. Nauss and coauthors reported epithelial ulceration and necrosis of the cornea of VAD rats inoculated with herpes simplex virus [11].

VA importance in reproduction and embryo development was demonstrated in studies showing that VAD female rats had difficulty becoming pregnant and that those that became pregnant produced pups with birth defects [50]. Previous animal studies have provided strong evidence that VAD is associated with adverse effects in offspring during the embryonic and post-natal period [49,51]. For example, El-Khashab and coauthors [52] showed that infants from VAD mothers had significantly lower mean retinol levels than their VAS counterparts, which was associated with fetal renal agenesis [53] due to smaller kidneys and decreased numbers of nephrons. VAD during pregnancy also hindered the development of pancreatic islet in rats and mice, an indication of a role of VAD in diabetes [54,55]. Additionally, breastfeeding by VAD mothers was shown to predispose infants to the effects of VAD. Moreover, prenatally acquired VAD in infants cannot be compensated by postnatal supplementation [54,56]. Therefore, maternal VAD not only affects maternal physiological and biological processes but also has a profound effect on the physiological and immunological development of the fetus, which persists postnatally. We demonstrated this effect in our previous studies in the Gn pig model where prenatally acquired VAD impaired both growth and immune development of neonatal piglets [56,57]. We have also shown that the effects of the prenatally acquired VAD on neonatal immune development could not be restored by postnatal VA supplementation. In the next section of this review, we present a detailed discussion of the immune impairment associated with VAD during infections and post-vaccination.

## 3. Vitamin A Deficiency Dysregulates Immune Function

Vitamin A regulates the immune response via very complex mechanisms involving the regulation of cytokine production to mount innate immune responses, mucosal barrier integrity and lymphocyte homing to mucosal surfaces [5] (Figure 2). Therefore, VA is a critical regulator of immunity against numerous infectious diseases and noninfectious health disorders [58]. VA regulates proliferation and function of NK cells [59,60] and also contributes to phagocytic and oxidative burst activity of macrophages [6,7,61]. NK cells play a vital role in the regulation of the early immune response to infections by producing proinflammatory cytokines (TNF-α, IFN-γ) that further regulate the function of other innate and adaptive immune cells [62]. Thus, impaired NK cell functions due to VAD compromise antiviral defense, which may limit other immune responses.

Previous studies have reported impaired functions of macrophages, neutrophils and NK cells and diminished protective function of the respiratory mucosa in VAD hosts [10]. Additionally, animal studies demonstrated that RA treatment of macrophages promotes an anti-inflammatory state, reducing proinflammatory cytokine IL-12 and TNF-α production and increasing anti-inflammatory cytokine IL-10 levels [63]. Spencer and coworkers using VAD mice demonstrated a reduction in the frequencies of type 3 innate lymphoid cells (ILC3s), cells responsible for IL-22 secretion, suggesting that ILC3 development and function are RA-dependent [64,65]. Hence, VA regulates production of IL-22, a key cytokine involved in the regulation of intestinal barrier function and homeostasis. IL-22 upregulates claudin-2 expression through the JAK/STAT pathway to increase intestinal epithelial permeability [66]. Tian and coworkers, using a mouse model of pneumonia, demonstrated that VAD mice had increased IL-12 and TNF-α levels resulting in their increased susceptibility to *Streptococcus pneumoniae*, a pathogen invading the mucosal epithelium [67]. In other studies using a rat model, VAD also increased macrophage-mediated inflammation via the enhanced production of IL-12 and IFN-γ and impaired phagocytic capacity of macrophages, leading to increased pathogen replication at the site of infection [11,12]. The enhanced secretion of these cytokines (due to both VAD and increased pathogen load) is known to exacerbate inflammation. For example, Wiedermann and coauthors observed that VAD decreased the phagocytic activity and bacteria-killing ability of peritoneal macrophages targeting *Staphylococcus aureus* in rats [12]. Additionally, VA has been shown to enhance tumor growth factor beta (TGF-β) secretion, which triggers IgA production by activated B cells and restores NK cell function essential for pathogen clearance [68,69].

VA induces the differentiation of naive T cells into regulatory T (Treg) cells through boosting Foxp3 gene expression and inhibiting Th1/Th17 generation [67,70,71,72,73]. Thus, VAD results in dysregulated adaptive immune responses by increasing CD8^+^ T cell numbers, IFN-α levels and proinflammatory IL-12 levels while reducing anti-inflammatory IL-10 levels [16]. VA through its biologically active metabolite all-trans-retinoic acid (ATRA) induces the expression of gut-homing receptors on lymphocytes [68,74]. Studies have shown that GALT CD103^+^ DCs enhance the expression of α4β7 and CCR9 on effector T cells through the RA-dependent pathway, thus enhancing protection against mucosal pathogens [75,76]. Previous studies in mice have demonstrated that T cell gut homing requires the expression of α4β7 integrin and CCR9 [74,75,77]. Additionally, RA is needed to induce gut-tropic IgA antibody (Ab) secreting cells (ASCs) in mice and humans. Mora and coauthors demonstrated the effect of VA on B cell homing and immunoglobulin class switching by incubating activated murine spleen B cells together with RA, which resulted in high levels of α4β7 and CCR9 expression on B cells [68]. Moreover, another group showed that RA can redirect immune responses elicited by the subcutaneous vaccination of mice from skin-draining inguinal lymph nodes to the gut [78]. RA is essential in the regulation of human polymeric immunoglobulin receptor (pIgR) expression in the presence of IL-4 and TNF-α, a mechanism that involves the RAR-dependent signaling pathway, leading to enhanced mucosal immunity [79,80]. Therefore, VAD could result in decreased sIgA levels due to reduced transport of mucosal IgA onto epithelial surfaces, and studies have shown that the administration of VA increased the concentrations of IgA in affected mice [13,14,15].

In summary, the bioactive VA metabolite RA is essential in the regulation of host immune responses, whereby it regulates the induction of lymphocyte homing to the gut and other mucosal surfaces, the production of IgA and the differentiation of Treg cells and Th17, as well as Th1 and Th2 polarization. Thereby, VAD has a profound effect on host immunity against numerous infectious and noninfectious diseases. In the next section, we review the current data on the effects of VAD on immune responses to selected infectious diseases and vaccines, summarizing the insights from clinical studies and animal model research.

### 3.1. Vitamin A Deficiency Increases Disease Severity

There is overwhelming evidence demonstrating a correlation between VAD and the severity of infectious diseases that lead to poor clinical outcomes in affected individuals (Table 1). Infections increase the host VA requirements, reduce the liver’s production of RBP [81] and reduce the intestinal absorption of VA due to impaired gastrointestinal mucosal functions [43]. Likewise, VA is an indispensable micronutrient that modulates Ab production and immune (B/T and NK) cells and maintains the integrity of the mucosal epithelial barrier, thus playing a critical role in anti-infectious defense [82,83]. VAD individuals with serum retinol levels of ≤0.70 µmol/L are vulnerable to certain enteric and respiratory infections, with younger children being at greatest risk [84,85]. On the other hand, VAS was shown to reduce mortality in preschool-aged children by reducing the severity of infectious diseases caused by VAD [86]. VAD leads to impaired epithelium integrity [87,88], which increases the risk of susceptibility to infections in infants [89]. Over the last three decades, there has been significant progress in understanding the role of VA in antimicrobial defense and its association with bacterial, viral and parasitic infections. To summarize, in the following sections, we will present a detailed discussion regarding the effects of VAD on the clinical outcomes of various infectious diseases in humans and animals.

#### 3.1.1. VAD Influences the Immune Response to Parasitic Infections While the Latter Modulate VA Metabolism and Bioavailability

Giardiasis is one of the most common causes of waterborne disease globally including in the United States. Giardia disrupts tight junctions, altering intestinal barrier integrity and leading to entry and spread of enteric pathogens [39]. The impaired expression of tight junction proteins has been correlated with the heightened secretion of IFN-γ, TNF-α and IL-13 (proinflammatory cytokines). *Giardia duodenalis* infection in a mouse model of human giardiasis was shown to induce the secretion of IL-22 in a CD4^+^ T cell-dependent manner [96]. Giardiasis has been implicated with VAD in causing the intestinal malabsorption of VA and the intense mobilization of liver retinol storage [97]. A randomized controlled trial performed in Brazil reported reduced total parasitic infections and *Giardia* spp. infections among children who were supplemented with VA compared with the placebo group, indicating the role of VA in improving host defenses against giardia infections. [98].

A cross-sectional study in a low-resource setting in Kenya reported a positive correlation between soil-transmitting helminth infection/*Ascaris lumbricoides* infections and VAD status in children [99]. The adverse effects of helminth infections on nutrition are known, including low appetite and nutrient intake, impaired absorption and increased nutrient loss or altered metabolism. Ascaris worms express RBPs, retinol dehydrogenases and RA receptors, which use retinol for growth and development [100]; hence, infection by *Ascaris lumbricoides* resulted in VAD. Furthermore, helminths are known to induce a Th2 cytokine response that leads to the increased production of anti-inflammatory cytokines such as IL-4, IL-5, IL-9 and IL-13, leading to the expulsion of GI parasites. Thus, helminth infections alter immune responses directly and indirectly via the modulation of VA levels and metabolism. Combined VAS and deworming have been shown to reduce Ascaris reinfection rates in infants; however, there is a need for further study to clarify the timing and duration of the combined interventions.

Visceral leishmaniasis causes hepatosplenomegaly and also affects the bone marrow and immune system. Lower serum retinol levels were observed in children with visceral leishmaniasis [101,102]. A cross-sectional study performed in Piauí, Brazil, to evaluate VA status with *Leishmania infantum* showed that VAD was associated with more severe leishmaniasis, and it was widespread among elderly populations; furthermore, VAD was associated with low hemoglobin and high C-reactive protein levels, leading to anemia development in VAD individuals [103]. Visceral leishmaniasis leads to intense cytokine release characterized by the increased production of IL-6, IFN-γ, IL-8, IL-1β and TNF-α, resulting in bleeding due to coagulopathy and thrombocytopenia [104]. On the other hand, Vellozo and coauthors demonstrated that RA intervention decreased resistance to Leishmania infection in mice, likely due to the fact that RA inhibits the development of M1 macrophages and protective type 1 immune cells and promotes the production of nonprotective M2 macrophages [105]. Similarly, VAD resulted in the dramatic expansion of the IL-13-producing type 2 ILC population and enhanced resistance to nematode infection in mice, further indicating the importance of ILCs as primary sensors of dietary stress [64]. In sharp contrast, Gundra and coauthors observed aggravation of the disease evident by excessive inflammation and tissue damage in VAD mice infected with *Schistosoma mansoni*, while RA administration enhanced the conversion of macrophages into a tissue-resident phenotype, diminishing inflammation and controlling the infection [106].

An impact of VAD on malaria morbidity was reported previously whereby malaria severity was associated with reduced serum retinol levels in rural Zambian children [107]. A randomized placebo/controlled trial evaluated the effects of VAS in a *Plasmodium falciparum* endemic area of Papua New Guinea [108] and demonstrated that VAS ameliorated malaria severity in young children. Similar findings were observed in rodent models [109,110]. Another randomized clinical trial conducted in Tanzania demonstrated that VAS ameliorated the adverse malaria effects on linear and ponderal growth in children [111]. The interactions between pro- and anti-inflammatory cytokines such as IL-12, IL-18 and TGF-β have been shown to play a key role in malaria pathogenesis and outcome, whereby they modulate the immune response against *Plasmodium falciparum*, which causes malaria [112,113]. The secretion of these cytokines is also influenced by the VA status of the host; hence, VAD may have a profound effect on malaria pathogenesis and outcome. VAS reversed the effects of VAD through the increased phagocytosis of nonopsonized red blood cells via the increased expression of CD36 cytoadherence receptors and reduced production of TNF-α via the downregulation of the peroxisome proliferator-activated receptor γ-retinoic X receptor [114]. Cryptosporidiosis, a diarrheal disease associated with VAD, causes intestinal damage, reducing villi and microvilli length and thereby resulting in the malabsorption of nutrients including VA [115,116,117]. Mucosal damage caused by cryptosporidium leads to the higher paracellular permeability of the intestine and a diminishing mucosal barrier function. Klein and coauthors, using a calf model, demonstrated that the impaired absorption of VA in the acute phase of cryptosporidiosis led to VAD [116]. In a triple cohort study, VAD in Haitian children was identified concurrently with acute cryptosporidiosis, proinflammatory Th2 (IL-8, TNF-α) and counterregulatory intestinal immune responses [118]. 

Carman and coauthors reported an association between *Trichinella spiralis* infection and VAD, where VAD mice had reduced frequencies of B lymphocytes, antigen-specific IgG1 Abs and eosinophils in the bone marrow [119]. Moreover, mesenteric lymph node cells from infected VAD mice produced significantly higher IFN-γ levels and fewer Th2 cytokines than *T. spiralis*-infected control mice (VAS) [119]. Similar observations were reported for *Toxoplasma gondii* infection in VAD mice [120]. Thus, inflammatory cytokines released in response to most parasitic intestinal infections likely contribute to the secretory response to these infections, and VA plays an essential role in defending the host against parasitic infections. In contrast, Kang and coworkers observed in a mouse model of Crohn’s disease that VAD decreased the homing of CD4+ T cells to the gut, which is beneficial during intestinal inflammation in inflammatory bowel disease (IBD) [121]. Whether this is true during GI parasitic infections is a question for further research. In conclusion, intestinal parasites (*Onchocerca volvulus*, *Brugia malayi*, *Ascaris suum*) secrete RBP that can sequester RA close to the parasite’s niche (immunomodulatory mechanism), depleting host RA (VAD) and ensuring parasite survival. Thus, the variation in immune responses to different intestinal parasites revealed a significant gap in the role of RA signaling during these infections, especially in VAD hosts, which requires further studies. 

#### 3.1.2. VAD Impacts Immune Response to Viral Infections

VAD results in increased susceptibility to viral infections and is associated with increased mortality through the modulation of the immune system, altering humoral and cellular immunity at mucosal surfaces [64,93,122,123].

Respiratory tract viral infections: VA plays a distinctive role in the respiratory tract, reducing inflammation, aiding in the repair of the respiratory epithelium and inhibiting fibrosis. Thus, VAD has been associated with the increased severity of multiple respiratory infections [41,95,124], where it causes squamous metaplasia of the respiratory epithelium: ciliated epithelial cells are replaced by squamous epithelium causing a decrease in mucus production. For example, Penkert and coworkers observed that VAD mice had prolonged upper and lower respiratory tract infection following Sendai virus (SeV) infection, which was associated with the increased production of proinflammatory cytokines/chemokines (IL-4, IL-5, IL-6, IL-12, IL-17, IFN-γ, TNF-α, MIP-1α, MIP-1β, MIP-2, LIX) in the nasal tissues of VAD mice [41]. Surman and coauthors demonstrated that virus-specific mucosal IgA responses were impaired in the respiratory tract of VAD mice infected with SeV [125,126,127]. The same group observed in their recent study altered T cell function in VAD mice infected with SeV as a result of low RANTES (regulated upon activation, normal t-cell expressed and secreted chemokine) levels (in addition to IL-1α and IL-5), while IFN-γ-induced protein 10 (IP-10) and eotaxin were increased, suggesting uncontrolled tissue damage in VAD mice [38]. Similarly, the same authors reported squamous cell metaplasia in the lungs of VAD mice. In addition, the partial loss of cilia and squamous metaplasia of the respiratory epithelium have been observed in VAD animals (mice and rats) with respiratory infections [40,82,88,128,129]. These abnormalities of the mucosal airways were shown to weaken pulmonary defenses against pathogens, hindering their clearance from the lungs and upregulating mucosal cytokines [41]. In humans, severe pneumonia caused by respiratory syncytial virus and/or human metapneumovirus was reported in hospitalized children below 5 years of age having low serum RBP [130,131], further supporting the role of VAD in enhancing the severity of respiratory viral infections. Additionally, Qi and coauthors reported an increased prevalence of acute respiratory tract infection and diarrhea in children with VAD [132]. VAD has also been associated with increased measles prevalence [133], and studies have shown that VAS reduced measles morbidity and mortality via increasing total leukocyte counts and reducing pro-inflammatory IFN-γ and Th1 responses [134,135]. In turn, measles aggravated VAD by reducing RBP synthesis, resulting in the increased excretion of VA in urine. Earlier studies showed that pediatric VAS resulted in shorter duration of illness and fewer complications caused by measles [134,136,137].

In the ongoing COVID-19 pandemic, damage to the mucosal barrier and immune cells due to the overproduction of pro-inflammatory (IL-1, IL-6, IL-12, IL-17, IFN-γ, TNFα) cytokines (cytokine storm) was evident in some COVID-19 patients [138]. Since VA is essential in the modulation of immune responses, there is a growing perception that VAS in COVID-19 patients may reduce the severity of infection [139,140]. Furthermore, the cellular mechanisms of SARS-CoV2 infection are controlled through the cell-surface receptor angiotensin-converting enzyme 2 (ACE2), whose expression has been shown to be regulated by ATRA [141]. Lung inflammation due to COVID-19 induced a decrease in RA uptake and the metabolism of polar metabolites, affecting the ability of lung to regenerate from pulmonary fibrosis [10,142].

In summary, VAD is associated with the enhanced severity of respiratory infections due to metaplasia of squamous cells and the loss of cilia in the airways, leading to the increased production of proinflammatory cytokines and reduced mucosal IgA responses as well as altered T cell responses. Supplementation of VA could rescue some (but not all) of these adverse effects of VAD and respiratory infections. Recent studies using calf and guinea pig models demonstrated that VA supplementation through inhalation is more efficient for addressing the immunological lesions in the VAD individuals [143,144].

Gastrointestinal tract viral infections: Increased frequencies of diarrheal episodes have been associated with increased VAD, and numerous studies have shown that VAS reduces the incidence of diarrhea in children below 5 years of age [95,145,146]. VA is critical for the intestinal immune response to enteric pathogens; as we discussed above for respiratory infections, VAD has profound effects on immune responses to gastrointestinal infections as a result of reduced MUC2 and elevated MUC3 expression, lower defensin 6 production and the upregulation of TLR2 and TLR5 expression [43]. Human rotavirus (HRV) causes a life-threatening viral gastroenteritis in infants and young children, especially in developing countries, and its increased prevalence and/or severity have been associated with VAD, where VAD aggravated the pathogenesis of HRV infection through increased intestinal damage [44,147]. Ahmed and coworkers demonstrated that VAD leads to the alteration of the small intestinal mucosa including damaged (blunted) tips of the villi of mice leading to decreased numbers of goblet cells [44]. Additionally, Raifen and coworkers observed smaller glandular areas and higher levels of MUC in the colon of VAD mice infected with rotavirus (RV), leading to higher viral shedding in their stool than in control mice [147]. Using a Gn piglet model, our lab previously showed that VAD caused a profound impairment of adaptive and innate immune responses, increasing the severity of HRV infection in the VAD piglets [16,56,57]. Specifically, VAD impaired innate immune response to HRV by reducing the numbers of conventional and plasmacytoid DCs while increasing the total numbers of undifferentiated immune cells of myeloid lineage [56]. The latter VAD-related phenomenon was also observed in a mouse model coinciding with the decreased expression of RAR and RXR nuclear receptors, as in our study [46,56]. Further, coincident with the increase in the numbers of undifferentiated mononuclear cells, IFN-α production was increased in VAD Gn piglets prior to HRV challenge but decreased following the HRV challenge [56]. Together, these observations are consistent with the VAD-mediated immune dysregulation and increased inflammation. In addition, there was a reduction in the frequencies of CD103 (integrin αEβ7) expressing DCs, suggesting an inhibitory effect of VAD on the imprinting of the gut homing phenotype by mucosal DCs [56]. We also observed elevated levels of the pro-inflammatory mediator IL-8 and decreased anti-inflammatory cytokine IL-10 levels in VAD Gn pigs after challenge with HRV coinciding with more severe inflammatory responses [57]. Moreover, we also demonstrated that high levels of IFN-α and IL-12 proinflammatory cytokines and CD8^+^ T cells and extremely low levels of IL-10 were associated with increased diarrhea severity after HRV challenge of the VAD piglets. The diarrhea severity was further associated with lower serum RV-specific IgA Ab titers and reduced numbers of intestinal HRV-specific IgA ASCs [16]. On the other hand, Cha and coauthors showed that VAD inhibited Th17 cell differentiation, resulting in low expression IRF4, IL-21, IL22, and IL-23 in the small intestines of mice [148].

Our recent study evaluating the effects of VAD on immune response to porcine epidemic diarrhea virus (PEDV), a highly contagious enteric coronavirus infection resulting in 80–100% mortality in piglets, demonstrated increased viral shedding titers and severe diarrhea in piglets born to VAD sows [92]. The severity of diarrhea in VAD pigs was correlated with decreased PEDV-specific IgA ASCs, IgA Ab titers in serum, and IgA+β7 B cells (gut homing) in milk. Similar to the observations in our VAD–HRV studies, there were increased levels of IFN-α and IFN-γ and decreased IL-10 and IL-22 levels in VAD sows following PEDV challenge [92]. Oral VAS to VAD sows restored these impaired immune responses, leading to enhanced lactogenic immune protection in the piglets [92].

The pathogenesis of another highly contagious enteric pathogen of humans and animals, norovirus (NoV), has also been linked to VAD effects. Long and coauthors [90,149] in their study of NoV in a cohort of children (in Mexico) reported divergent impacts of VAS on the immune responses to NoV genogroup (NoV GI and NoV GII) infection, where they observed a reduction in monocyte chemoattractant protein-1 (MCP-1), TNF-α, IL-6, and IL-8 levels among VAS children with NoV GII-associated diarrhea, while observing higher TNF-α and IL-4 levels among VAS children with NoV GI-associated diarrhea [90]. Lee and Ko demonstrated that VA mediated its effects on NoV infection via the modulation of the gut microbiota, activating IFN (IFN-β and -γ) signaling by VA and increasing *Lactobacillus* spp. in response to murine NoV infection using a mouse model [150].

Hepatic and neurological viral infections: Previous studies have reported an association between lower serum retinol levels (VAD) and the increased severity of hepatitis C virus (HCV) infection [151,152,153]. The low serum VA levels were associated with reduced levels of circulating RBP4, a VA transporter, as a result of liver damage. The reduction of VA, an antioxidant, may also be linked to greater body physiological requirement due to the oxidative processes, leading to an imbalance in the cellular redox balance [154]. Moreover, VAD was associated with HCV chronic infection and with unresponsiveness to interferon-based antiviral therapy [151]. Additionally, Bocher and coauthors reported a strong anti-HCV and synergistic effect of ATRA and pegylated IFN-α2a treatment leading to reduction in HCV replication in vitro [155].

Studying neurological disease using a mouse model of persistent lymphocytic choriomeningitis virus (LCMV), Liang and coauthors demonstrated that VAD causes abnormal (hyperreactive) T cell response to viral infection, leading to intensified tissue damage and mortality in mice [45]. The infected VAD mice showed low levels of programmed death-1 (PD-1), a marker of germinal center-associated T cells and angioimmunoblastic T-cell lymphoma, and elevated proinflammatory cytokine (TNF-α, IFN-γ, and IL-2) production by T cells. Importantly, Liang and coauthors demonstrated that VAS modulated immune responses and protected VAD mice from persistent LCMV. Moreover, VAS mice had reduced nuclear factor of activated T cells 1 (NFATc1), a key regulator of T cell activation [45].

In summary, these data suggest that VAD leads to the profound dysregulation (not just suppression) of local and systemic antiviral immune responses favoring pro-inflammatory reactions.

#### 3.1.3. VAD Impairs Immune Response to Bacterial Infections

Recently, Penkert and coauthors demonstrated impaired immune responses to influenza virus in VAD mice and consequently increased susceptibility to secondary *Streptococcus pneumoniae* coinfection [129]. This was due to a malfunction in CD4^+^ T-cell recruitment and B-cell organization in the lungs of the VAD mice, resulting in elevated levels of IFN-β, IFN-γ, and IL-1β and the increased recruitment of neutrophils, eosinophils, and macrophages into the lung. Moreover, the VAD mice exhibited exacerbated inflammation and epithelial hyperplasia [129]. Recently, Xing and coworkers reported an association between VAD and severe *Mycoplasma pneumoniae* pneumonia in children [91], which was due to the increased infiltration of proinflammatory cells, and the hyperpermeability of airway epithelial cells [156]. Furthermore, Tian and coauthors observed that VAD triggered asthma in mice following *Streptococcus pneumoniae* infection [67], while VA administration to VAD mice substantially increased Foxp3^+^ Treg cells and IFN-γ but reduced IL-4, IL-5, and IL-13 cytokine production and Th17 cell numbers, thereby alleviating airway hyperresponsiveness and inflammatory cell infiltration during allergic airway infection. Thus, VAS hampered the progression of asthma by altering CD4^+^ T cell subsets in neonatal mice [67]. Moreover, the severity of Hansen’s disease (leprosy) caused by *Mycoplasma leprae* was positively associated with VAD [157].

Vitamin A affects tuberculosis (TB) disease development and progression, as has been observed among pulmonary TB patients in Lima, Peru [158], where VAD greatly increased the risk of TB, while VAS provided an effective way of preventing it. Additionally, a case–cohort study of HIV-positive adults from nine different countries reported an association of VAD with TB and recommended VAS to reduce TB in high-risk HIV-positive patients [159,160]. Greenstein and coworkers observed a dose-dependent inhibition of *Mycobacterium tuberculosis* growth in culture after VA treatment [161]. Research has shown that VA (ATRA) mediated antimicrobial effects against *M. tuberculosis* via modulating cellular cholesterol efflux through the Niemann–Pick C intracellular cholesterol transporter 2 (NPC2) gene. In addition, the impact of RA on HIV replication correlated with the cellular expression of RA receptors, where HIV-positive patients had consistently lower serum retinol levels [162,163,164,165]. Moreover, the administration of VA reduced the mortality and morbidity among HIV-positive children due to a profound increase in total lymphocyte counts and CD4^+^ T-cells [166], although there was a limited effect of VAS in HIV-positive adults [1,167,168].

Studies have also associated VAD with *Chlamydia trachomatis* infection in children [169], where it was reported that VAD resulted in the loss of goblet cells that led to trachoma [169,170]. Cantorna et al. also observed that VAD aggravated acute Lyme arthritis in *Borrelia burgdorferi*-infected mice that was associated with high production of IL-12 and IFN-γ [171]. RA is a known inhibitor of IL-12 and IFN-γ transcription suggestive of a correlation between VAD and milder arthritis. *Borrelia burgdorferi* causes Lyme disease in humans; hence, we hypothesize that VAD may exacerbate the severity of Lyme disease in a population where VAD is prevalent. 

Yang and coauthors reported the impact of VAD on mucosal immunity against intestinal infection by *Salmonella typhimurium*, where they observed impaired humoral and cellular immunity in the intestinal mucosa due to markedly upregulated mucosal dendritic cells, IL-12, TLR2, MyD88 and IFN-γ but decreased intestinal sIgA in Gn rats [93]. On the other hand, during salmonella infection, the host increases IL-22–mediated antimicrobial factor (RegIII-γ and calprotectin) production in the gut, and since salmonella is resistant to these antimicrobial factors, it thrives while the beneficial commensals perish. Thus, commensal species evolved mechanisms to inhibit RA synthesis and limit the ability of salmonella to hijack the host immune response. McDaniel and coauthors observed that VAD mice developed a severe and lethal *Citrobacter rodentium* infection that was associated with reduced frequencies of T cell receptor (TCR), TCRβ^+^, TCRβ+/CD8α^+^ and TCRβ^+^/CD8αβ^+^ T cells [94]. In agreement with the previous studies [93,172], He and colleagues also reported an association between VAD and *E. coli* infection in feedlot cattle where they observed that VAD cattle were more susceptible to *E. coli,* presenting intractable diarrhea compared to the control animals [173].

Taken together, VA is essential for immune responses against numerous infectious diseases; hence, its deficiency has profound effects on the tri-directional interactions between VA, immune responses and infections. From the discussion above, it is evident that VA supplementation is capable of reversing many, but not all, of the VAD-induced adverse effects on antimicrobial immunity. Therefore, programs that enhance dietary VA intake in populations where VAD is prevalent are essential to reducing its prevalence and to maintaining/restoring immune functions, physical barrier integrity and protective mechanisms against infectious diseases in these populations. Therefore, assessing VA as a therapy during infection in malnourished hosts may lead to improved health outcomes. However, the dichotomy of the VAD effects observed in antiviral/antibacterial vs. antiparasitic infections and immunity has to be carefully considered and evaluated in dually infected animal models. In the next section, we present a detailed discussion on how VAD influences the immune response to vaccine antigens.

### 3.2. Vitamin A Deficiency Influences Immune Responses to Vaccines

Vaccination is the best weapon and most cost-effective tool for protection against infectious diseases in humans and animals. Vaccines induce or boost protective immunity by mimicking natural infection and causing the immune system to produce antigen-specific T-lymphocytes, antibodies (Abs) and other antimicrobial factors. As we have detailed in the previous sections, VA is essential in the regulation of immune responses to infections; hence, VA is also critical for the modulation of the immune response to vaccines. Therefore, in this section, we review current literature regarding the effects of VAD on the immune responses to vaccines. A list of selected clinical and animal studies investigating the effects of VAD on the immune response to vaccination is presented in Table 2. Previous studies have reported an association between VAD and poor response to vaccination, leading to the increased risk and severity of various diseases and mortality [174,175].

Studies using murine models observed ineffective Ab production and decreased numbers of ASCs and T-cells in VAD mice following vaccination against SeV infection [125,176]. Penkert and coworkers, using a mouse model of influenza infection, demonstrated that VAS significantly enhanced virus-specific Ab (IgM/IgG) responses, improved the lung immune environment (increased CD4^+^/CD8^+^ T cells) and reduced inflammatory cytokine (IL-12, IP-10, MCP-1) levels and viral loads after vaccination and post-virus challenge in VAD mice [177]. Similarly, Surman and coauthors observed reduced IgA-producing ASCs in VAD mice following intranasal influenza vaccination [127,178], suggesting that VA administration to VAD mice enhanced these mucosal IgA Ab responses post-vaccination. Moreover, Penkert and coworkers demonstrated that when VA was administered to VAD mice concurrently with the pneumococcal (Prevnar-13) vaccination followed by challenge with *S. pneumoniae*, the levels of pneumococcus (T4)-specific Abs were elevated, leading to increased survival rates in the VA supplemented VAD mice [179]. Using a mouse model of tetanus disease, Molrine and coauthors demonstrated significantly reduced anti-tetanus toxoid Ab production in VAD mice compared with VAS mice following vaccination against tetanus and also that VA administration to VAD mice enhanced Ab production post vaccination [180]. Kaufman and coworkers demonstrated that moderate VAD rescinded antigen-specific CD8^+^ T cells trafficking to the gut, gastrointestinal cellular immune responses and protection against a mucosal challenge following mouse immunization with a recombinant adenovirus vaccine vector [181]. However, oral VA administration completely restored the mucosal immune responses, upregulating α4β7 integrin and thus enhancing vaccine efficacy.

McGill and coauthors using a calf model of respiratory syncytial virus (RSV) infection reported that VAD led to the impairment of the immune response to intranasal vaccination against bovine RSV infection in neonatal calves [143]. These authors showed that vaccinated VAD calves failed to mount virus-specific IgA/IgG Ab responses in the bronchoalveolar lavage. Moreover, the expression of IL-6, IFN-γ, IL-13, CXCL9, CXCL10 and TGF-β was significantly elevated in the lungs of VAD vs. VAS calves, promoting a pro-inflammatory microenvironment. In contrast, the expression of IL-17 and the MUC5B mucin gene were both significantly decreased in VAD calves compared with VAS calves [143].

Clinical studies in the US and Turkey demonstrated that children with VAD had lower measles-specific Ab levels than their control counterparts after vaccination [124,182]. Benn and coworkers also reported enhanced measles virus-specific IgA Ab levels in VAS males compared with VAD males in Guinea-Bissau, west Africa [183], while Jensen and coauthors observed increased leukocyte counts and IFN-γ secretions in females who received VAS concurrently with measles vaccine in the same country [135]. Similarly, Rahman and coauthors also demonstrated that supplementing VA concurrently with routine immunization increased Ab responses to the diphtherial vaccine in infants younger than 6 months in Bangladesh [174]. A study from Ghana demonstrated an enhanced immune response to the hepatitis B vaccine when VA was administered to infants concurrently with the pentavalent ‘diphtheria-polio-tetanus-*Haemophilus influenzae* b-hepatitis B vaccine [184]. However, VA administration did not affect the immune response to *Haemophilus influenzae* type b vaccine [184]. Previous clinical studies in Zimbabwe and Indonesia also showed that oral VAS did not affect Ab responses to poliovirus vaccine in infants [185,186], suggesting that the mechanisms through which VAD affects the immune response to vaccination may depend on the vaccine/pathogen type.

Our laboratory, using a Gn piglet model for HRV infection and monovalent HRV vaccine, observed higher serum IFN-α, IL-12 and CD8^+^ T cells in the blood and intestinal tissues and reduced IL-10 in unvaccinated VAD piglets post-HRV challenge, coinciding with higher diarrhea severity and fecal shedding [16]. Moreover, VAD piglets immunized with monovalent HRV vaccine had increased diarrhea severity post-HRV challenge that coincided with augmented IL-12 and IFN-γ secretion, reduced serum HRV-specific IgA Ab titers and intestinal HRV-specific IgA ASCs and decreased the frequency of Foxp3^+^ Treg cells among systemic and intestinal mononuclear cells [16]. Similarly, using the Gn pig model of HRV infection and pentavalent HRV vaccine, we demonstrated that vaccinated VAD piglets had greater fecal virus shedding, coinciding with increased IL-8 levels and reduced HRV-specific IgA Ab titers and IL-10 levels compared with vaccinated VAS piglets post-HRV challenge [57]. Furthermore, vaccinated VAD piglets had significantly reduced ileal HRV-specific IgG and duodenal HRV-specific IgA ASC numbers pre-challenge and post-HRV challenge, respectively, compared with vaccinated VAS piglets, leading to increased diarrhea severity in vaccinated VAD piglets. Additionally, increased IFN-γ levels were observed in VAD piglets pre-challenge, suggestive of enhanced pro-inflammatory immune responses [57]. Studying the effects of prenatally acquired VAD on innate immune responses to HRV infection and vaccine in the Gn piglet model, we observed impaired innate immune responses and severe diarrhea in VAD piglets; however, postnatal VAS failed to restore the impaired innate immune responses [56]. This study showed that regardless of the vaccination status, the numbers of conventional/plasmacytoid DCs were greater in VAD piglets pre-challenge, but these cells were significantly reduced together with CD103 (integrin αEβ7)-expressing DCs in VAD piglets post-HRV challenge [56]. The latter likely led to suboptimal T and B cell responses systemically and in the gut. Finally, VAD piglets exhibited greater virus shedding titers and serum IFN-α levels at 2 days post-HRV challenge; however, IFN-α levels together with TLR3+ expressing MNCs were reduced at 10 days post-HRV challenge.

In summary, the clinical and animal model studies showed that VAD has overwhelming effects on the immune responses elicited by vaccines. Moreover, some studies have revealed that VAS before or during immunization against some infectious diseases did not restore the impaired immune responses. Analysis of the existing evidence and data suggests that the ‘one-size-fits-all’ approach of VAS to improving immune responses and protection against multiple pathogens is not feasible. Therefore, programs that recommend VAS during immunization to boost vaccine immunogenicity should be evaluated further because the effects may be specific to each vaccine type, VA supplementation regimen (single high dose vs. continuous small doses), timing (maternal/prenatal vs. postnatal/neonatal) and even administration route.

## 4. Gut Microbiota Adaptation to Vitamin A Deficiency Impacts Host Immunity

The gut microbiota plays a vital role in the development of lymphoid structures and regulates the different functions of immune cells [187,188,189,190]. Moreover, the immune system is also essential for host control over microbiota composition, ensuring the maintenance of the homeostasis of the host–microbe relationship [191] (Figure 1). Studies have shown that the microbiota contributes to the repair of the damaged intestinal epithelium through an MyD88-dependent process, a process that also stimulates the production of antimicrobials such as α-defensins, preventing the colonization of the gut by pathogens [192,193,194]. Furthermore, an essential defensin, cathelicidin (LL-37), is synthesized in Paneth cells under the control of vitamins A and D [195], suggesting a synergistic effect of VA and the gut microbiome in the prevention of colonization of pathogenic microbes. Additionally, the gut microbiome inhibits pathogen growth by producing high amounts of short-chain fatty acids (SCFAs), a vital energy source for intestinal epithelial cells, which helps to strengthen the mucosal barrier and modulates immune responses [196,197]. 

Dietary VA status has a substantial influence on normal state of host–microbiota interactions and thus susceptibility to infections (Table 3). Levy and coauthors showed that the gut microbiota (both commensal and pathobionts) immensely influences VA metabolism in the host, and that deficiency of VA impairs microbiota composition and immune function [198]. A recent study by Bonakdar and coauthors demonstrated that gut commensal bacteria produced high concentrations of ATRA, 13-cis-retinoic acid (13cisRA) and retinol [199]. The authors showed that the ablation of anaerobic bacteria significantly reduced concentrations of these retinoids, whereas introducing these bacteria into germ-free mice significantly enhanced retinoids. In addition, the same authors using in vitro assays demonstrated that gut bacteria modulate VA metabolism independent of the host, where they observed that *Lactobacillus* spp. dominates cultures supplemented with RE and retinal.

Inflammatory immune responses in the gut, including those associated with VAD, change the gut luminal environment which may favor dysbiosis. Hibberd and coauthors demonstrated that acute VAD causes a significant change in the microbiome structure (especially an increase in *Bacteroides vulgatus*) and their meta-transcriptome in the human gut microbiota–associated mice [200]. Grizotte-Lake and coworkers showed that Gn mice expressed elevated RA-synthesizing enzyme levels in their intestine compared with conventional mice and that the colonization of the gut by microbes reduced RA levels in the intestine [201]. The gut microbiota prevents the colonization and growth of pathogens by inducing the secretion of antimicrobial peptides and cytokines (IL-22, IL-17 and IL-10) while stimulating inflammasome activation. On the other hand, VA through its metabolite RA alters the frequency of ILC3s and Th17 T cells recruited into the gut, influencing IL-22 production, a vital cytokine required for pathogen clearance. This was demonstrated in a mouse model whereby VAD mice were vulnerable to *Citrobacter* infection [64,94]. Recently, Chen and coauthors showed that VAD exacerbates gut microbiota dysbiosis in transgenic mice [8].

Previous studies have also reported that VAD alters the bacterial population (especially low abundance of *Lactobacillus* spp.) and innate immunity-related genes in the gut of VAD rats [43], whereby VAD significantly changed mucin dynamics with a reduction in MUC2 in the small intestine and a rise in MUC2 in the large intestine. Similarly, Choudhury and coworkers demonstrated that the commensal bacteria burden in VAD rats was increased due to the decrease in MUC2 gene expression [202].

Lee and Ko reported that VA modulates the gut microbiome (increasing abundance of bacteria of *Lactobacillaceae* families), which upregulated the IFN-β response and prevents norovirus (NoV) replication [149,150]. Similarly, *Lactobacillus* spp. have been shown to exhibit antiviral effects against RV and influenza infections [203,204,205]. Cha and coauthors, using a mouse model, demonstrated that the disruption of gut microbiota (reduction of segmented filamentous bacteria, SFB) due to VAD resulted in the downregulation of Th17 cells in the small intestinal lamina propria, which promoted pathogen colonization [148,206]. Studies of VAD effects on microbiome diversity at different stages of life in rats demonstrated an alteration of the microbial diversity in the colon leading to an imbalance in *Firmicutes* and *Bacteroidetes* ratio [207,208].

Scott and coauthors using a mouse model of Alzheimer’s disease (AD) showed that the gut microbiota composition is controlled by diet (specifically VA), leading to the secretion or accumulation of amyloid protein in the brain, thus concluding that the gut–brain axis may be essential in the process of VAD exacerbating AD [209,210]. Moreover, using a mouse model of AD, Chen and coworkers also confirmed that VAD aggravates gut microbiota dysbiosis, which lowers γ-aminobutyric acid (GABA) receptor expression and downregulates the brain-derived neurotrophic factor (BDNF) in the brain cortex [8]. The gut microbiota’s role in the bidirectional signal transduction through the gut-brain axis has also been reported in other studies [211,212,213,214].

**Table 3:** Clinical studies and animal model research on interactions between VAD, gut microbiota and impaired immune responses to infectious diseases and vaccines.

Host Model	Pathogen/Study	Key Findings	Reference
Rat	Gut microbiome	A decrease in proportion of *Lactobacillus* spp. in VAD rats led to decreased MUC2 mRNA expression and increased MUC3 mRNA expression in intestine.	[43]
Rat	Gut microbiome	*Faecalibacterium* abundance was positively correlated with, while *Staphylococcus* abundance was negatively correlated with, serum retinol levels.	[207]
Mouse	Gut microbiome	VAD causes a significant change in the microbiome structure and its meta-transcriptome (increase in *Bacteroides vulgatus*) in human gut microbiota–associated mice	[200]
Mouse	Rotavirus/Influenza virus	*Lactobacillus* spp. have been shown to exhibit antiviral effects against RVand influenza infections.	[205]
Mouse	Gut microbiome	VAD reduced gut microbiome diversity by increasing expression of MUC2 gene by goblet cell hyperplasia, resulting in low IL-17, IRF4, IL-21, IL-22 and IL-23.	[148]
Mouse	Alzheimer’s disease Gut microbiome	VAD aggravates gut microbiota dysbiosis and cognitive deficits, reduces the expression of GABA receptors and downregulates BDNF in the brain through intestinal microbiota disruption.	[8,209]
Mouse	Norovirus Gut microbiome	The abundance of *Lactobacillus sp* inhibited murine NoV proliferation via the upregulation of IFN-β; RA administration increased their abundance levels.	[150]
Human	Gut microbiome polio virus, tetanus toxoid, hepatitis B virus vaccines	Bacterial diversity and abundance of Enterobacteriales, Pseudomonadales and Clostridiales were associated with neutrophilia and lower vaccine responses.	[9,215]
Human	Cholera vaccine	High abundance of *Clostridiales* in responders, while poor vaccine response was associated with *Enterobacterales*	[216]
Human	Rotavirus vaccine	Negative association between vaccine immunogenicity and gut microbiota diversity.	[217,218]
Pig	Influenza A virus	Influenza vaccine is more effective in pigs with *Prevotella* colonization than *Helicobacter* and *Bacteroides* colonization.	[219]
Chicken	*Campylobacter jejuni*/Vaccine	Abundance of *Clostridium* spp., *Ruminococcaceae* and *Lachnospiraceae* was positively associated with vaccine-induced antigen-specific IgY responses.	[220]

Numerous studies have reported on the association between the gut microbiota composition and vaccine immunogenicity [221,222,223,224]. Some of the gut microbiota members shown to have immunomodulatory roles include *Bacteroides fragilis*, *Clostridia* spp. and SFB [225,226,227,228,229,230,231,232]. Harris and coauthors reported a positive correlation between oral HRV vaccine immunogenicity with dominant members of *Bacilli* phylum and increased *Enterobacteriaceae*-to-*Bacteroidetes* ratio among Ghanian children [233]. Similarly, in Pakistani children, an abundance of Gram-negative bacteria such as *Serratia* and *E. coli* correlated positively with immune responses to HRV vaccine [233]. Other microbe abundances reported to be positively correlated with vaccine immunogenicity include *Bifidobacterium longum* vs. polio, Bacillus Calmette-Guérin and tetanus vaccines [215], *Clostridiales* vs. *Salmonella Typhi*/cholera vaccine [216,234] in humans; *Collinsella*/*Lactobacillus* LGG/*Bifidobacterium* Bb12 vs. RV vaccine [61,235,236], *Prevotella* vs. influenza A vaccine [219] in pigs; *Clostridium* spp., *Ruminococcaceae*, *Lachnospiraceae* vs. *Campylobacter jejuni* [220] in chickens. Therefore, it is well established that perturbations of the gut microbiome by either antibiotics or dietary (VAD) can have major impacts on vaccine immunogenicity.

Taken together, the reviewed literature confirms that the microbial regulation of VA metabolism influences infection, while infection aggravates VAD through the reduction of VA absorption. Furthermore, the immune system affords its host certain control over the composition of their commensal microbiome, but these commensals can be perturbed by VAD-induced defects in the immune system, thereby profoundly impacting the host’s health. Therefore, a deeper understanding of molecular mechanisms underlying these interactions is essential for designing effective strategies to improve human and animal health.

## 5. Concluding Remarks

Vitamin A deficiency is not fully restricted to low-resource countries but has worldwide prevalence and is one of the nutritional problems of upmost importance together with iron deficiency and protein–calorie malnutrition. However, VAD cases in the developed nations are typically due to primary and secondary intestinal malabsorptive pathologies (including celiac disease, liver cirrhosis, pancreatic insufficiency, bile duct disorders) rather than low dietary intake. VAD due to low dietary intake is also found in low-income settings of developed nations. VAD is common in children less than 5 years of age and accounts for about 2% of deaths in this age group. Pregnant and lactating women are also at high risk of VAD due to increased daily nutritional requirements. In this review, we have summarized that VAD has profound effects on immune responses to infectious diseases and vaccines where it exacerbates intestinal inflammation, elevates redox stress and reactive oxygen species levels, impairs innate and adaptive immunity, compromises mucosal barrier functions and causes gut dysbiosis and/or loss of vaccine efficacy. However, there are still major gaps in our understanding of the multidirectional relationships between VA, the gut microbiome, the immune system and infection since some studies have reported the lack of or negative impacts of VAS on immune response to some infections/vaccines. The differential effects of VA supplementation may be dependent on the nutritional status of the host, the gut microbiome composition of the host, the most recent vaccines given, the sex and age of the individual, the season or the differential effects of RA on target cells. The findings imply that additional in vitro and in vivo studies are needed to clarify the mechanisms of VA/RA action, counteract VAD effects on immune responses to infections and vaccines and identify host-specific bacterial species associated with improved VA metabolism. Moreover, current immunization programs that recommend VA administration together with vaccine to boost vaccine efficacy should take the above variables into consideration. Overall, improvements in vaccine effectiveness may ultimately reduce the morbidity and mortality caused by infectious diseases worldwide. Future research seeking treatments for inflammatory infectious diseases should consider the role of VA in immune homeostasis and infection susceptibility.

## Figures and Tables

**Table 1:** Effects of VAD on immune responses to infectious diseases observed in individual clinical studies and animal model research.

Host Model	Pathogen	Key Findings	Reference
Human	Norovirus	VAS reduced MCP-1, IL-8, TNFα and IL-6 fecal levels in children with GII-associated diarrhea. However, VAS increased TNFα and IL-4 levels in children with GI-associated diarrhea.	[90]
Human	*S. pneumoniae*/Asthma	VAS after neonatal *S. pneumoniae* pneumonia enhanced Foxp3^+^Treg and Th1 secretion, decreased Th2 and Th17 cell expressions and lessened airway hyperresponsiveness and inflammatory cell infiltration during asthma.	[67]
Human	*Mycoplasma pneumoniae* pneumonia (MPP)	Both severe MPP (sMMP) and nonsevere MPP (nsMMP) led to VAD; however, VA levels were lower in the sMPP than nsMPP serum.	[91]
Gnotobiotic pig	Rotavirus (RV)	More severe prolonged diarrhea and increased human RV (HRV) fecal shedding post-challenge in VAD pigs coinciding with lower HRV specific IgA and Abs in serum and intestinal contents reduced HRV-specific IgA ASCs in ileum and duodenum pre- and post-challenge, increased levels of pro-inflammatory cytokine (IL-12) and CD8^+^ T cells in blood and intestines and lowered anti-inflammatory cytokine (IL-10). Higher numbers of DCs in VAD piglets prechallenge, but these cells decreased markedly postchallenge. Lower frequency of CD103 (integrin αEβ7) expressing DCs. Elevated necrotic MNCs in spleen prechallenge and in intestinal tissues postchallenge.	[16,56,57]
Pig	Porcine Epidemic Diarrhea Virus (PEDV)	VAS increased PEDV IgA ASCs and Abs in serum pre-partum and IgA^+^β7^+^ (gut homing) B cells in milk post-piglet PEDV challenge.	[92]
Rat	*Salmonella typhimurium*	VAD increased the number of mucosal DCs, IL-12, TLR2 and MyD88 while decreasing IFN-γ and sIgA production.	[93]
Mouse	RV	VAD reduced the number of goblet cells per duodenal villi. RV infection caused complete destruction of the villi in VAD mice. Thus, although RV infection and VAD cause few changes alone, together they led to complete damage of the gut mucosal barrier.	[44]
Mouse	Enteric infections	Lower T cell frequencies in the gut of VAD mice. *C. rodentium* infection aggravated inflammation and epithelial hyperplasia in VAD mice; however, RA treatment led to clearance of *C. rodentium*.	[94]
	gastrointestinal/respiratory tract infection	Every 10 mg/dL plasma retinol was associated with 18% fewer days of diarrhea with vomiting, 10% fewer days of cough with fever and 6% fewer doctor visits.	[95]
Mouse	lymphocytic choriomeningitis virus	VAD mice showed extreme inflammation and more severe liver pathology, with high deaths during persistent infection. Infected VAD mice had decreased PD-1 but increased cytokine (IFN-γ, TNF-α and IL-2) expression by T cells.	[45]

VA—Vitamin A; VAS—Vitamin A supplementation; VAD—Vitamin A Deficiency.

**Table 2:** Effects of VAD on immune responses to vaccination observed in individual clinical studies and animal model research.

Host Model	Pathogen	Key Findings	Reference
Human	Norovirus	VAS reduced MCP-1, IL-8, TNFα and IL-6 fecal levels in children with GII-associated diarrhea. However, VAS increased TNFα and IL-4 levels in children with GI-associated diarrhea.	[90]
Human	*S. pneumoniae*/Asthma	VAS after neonatal *S. pneumoniae* pneumonia enhanced Foxp3^+^Treg and Th1 secretion, decreased Th2 and Th17 cells expressions, lessened airway hyperresponsiveness and inflammatory cell infiltration during asthma.	[67]
Human	*Mycoplasma pneumoniae* pneumonia (MPP)	Both severe MPP (sMMP) and non-severe MPP (nsMMP) led to VAD; however, VA levels were lower in the sMPP than nsMPP serum.	[91]
Rat	*Salmonella typhimurium*	VAD increased the number of mucosal DCs, IL-12, TLR2 and MyD88 while decreasing IFN-γ and sIgA production.	[93]
Mouse	Sendai virus	The VAD mice showed aberrant serum Ig isotypes (high IgG2b levels) and cytokine/chemokine patterns (elevated eotaxin), with high frequencies of nephropathy and death.	[38]
Mouse	Rotavirus (RV)	VAD reduced the number of goblet cells per duodenal villi. RV infection caused complete destruction of the villi in VAD mice. Thus, although RV infection and VAD cause few changes alone, together they led to complete damage of the gut mucosal barrier.	[44]
Mouse	Enteric infections	Lower T cell frequencies in the gut of VAD mice. *C. rodentium* infection aggravated inflammation and epithelial hyperplasia in VAD mice; however, RA treatment led to clearance of *C. rodentium*.	[94]
	gastrointestinal/respiratory tract infection	Every 10 mg/dL plasma retinol was associated with 18% fewer days of diarrhea with vomiting, 10% fewer days of cough with fever and 6% fewer doctor visits.	[95]
Mouse	lymphocytic choriomeningitis virus	VAD mice showed extreme inflammation and more severe liver pathology, with high deaths during persistent infection. Infected VAD mice had decreased PD-1 but increased cytokine (IFN-γ, TNF-α and IL-2) expression by T cells.	[45]
Pig	Porcine Epidemic Diarrhea Virus (PEDV)	VAS increased PEDV IgA ASCs and Abs in serum pre-partum and IgA^+^β7^+^ (gut homing) B cells in milk post piglet PEDV challenge.	[92]
Gn pig	Rotavirus (RV) (human RV vaccines)	Increased levels of IL-8, a pro-inflammatory mediator and decreased IL-10 responses (anti-inflammatory) in vaccinated VAD compared with VAS piglets indicating more severe inflammatory responses in vaccinated VAD piglets post-challenge. Vaccinated VAD pigs had lower serum IgA HRV Ab titers and significantly lower intestinal IgA ASCs post-challenge suggesting lower anamnestic responses	[16,57]

VA—Vitamin A; VAS—Vitamin A supplementation; VAD—Vitamin A Deficiency.

## Data Availability

Not applicable.
